# How issue frames shape beliefs about the importance of climate change policy across ideological and partisan groups

**DOI:** 10.1371/journal.pone.0181401

**Published:** 2017-07-20

**Authors:** Shane P. Singh, Meili Swanson

**Affiliations:** 1 Department of International Affairs, University of Georgia, Athens, Georgia, United States of America; 2 Sandia National Laboratories, Albuquerque, New Mexico, United States of America; University of Vermont, UNITED STATES

## Abstract

We use an experiment to examine whether the way in which climate change is framed affects individuals’ beliefs about its importance as a policy issue. We employ frames that emphasize national security, human rights, and environmental importance about the consequences of climate change. We find no evidence that issue frames have an overall effect on opinions about the importance of climate change policy. We do find some evidence that the effect of issue frames varies across ideological and partisan groups. Most notably, issue frames can lead Republicans and those on the political right to view climate change policy as less important. We conclude by discussing our findings relative to extant literature and considering the implications of our findings for those who seek to address the issue of climate change.

## Introduction

In December 2015, over 120 world leaders attended the United Nations Climate Change Conference, where a historic international agreement on climate change was reached. The agreement affirms that climate change presents an “urgent and potentially irreversible threat” to humans and Earth, thus calling for global cooperation to address it [1]. An earlier United Nations report recognizes that climate change needs to remain relevant among competing foreign policy issues in order for the international community to successfully develop and implement global climate change policies [2]. Despite international acknowledgment of climate change as a major concern, a 2014 Gallup Poll found that, when presented with a list of 15 issues, Americans ranked climate change as one of the least worrisome [3]. Moving forward, a key challenge facing countries will be to mobilize adequate domestic support for policies aimed at climate change mitigation. In the United States, in particular, climate change is heavily politicized, making it difficult for both ordinary individuals and policymakers to reach a consensus on how to appropriately address the issue. This dilemma raises the question: can the issue of climate change be constructed in a way to increase the importance individuals assign it?

In his 2014 James Madison Lecture at the Annual Meeting of the American Political Science Association, Robert Keohane discussed how the framing of climate change by politicians and governments can affect policy responses to the issue and, in turn, the likelihood of effective action [4]. Policymaking is, of course, also influenced by public opinion [5, 6], and there is robust evidence that public opinion varies according to how an issue is presented [7–9]. Extant work finds that public opinion about climate change, specifically, can vary according to the way in which the issue is put forth [10–12]. Such variation likely influences the character of public policy.

A number of studies use randomized experiments to isolate the effects of issue framing on public opinion about climate change. Yarnal et al. [13] find that attitudes toward the way in which climate change should be addressed vary according to whether it is framed as a local or national issue. Schuldt et al. [14] find that using the term “global warming” rather than “climate change” can affect the probability that individuals perceive the phenomenon as real. Petrovic et al. [15] find that alluding to man-made climate change decreases the likelihood that conservatives agree that air pollution is harmful. Aklin and Urpelainen [16] find that framing climate change as a matter of national or economic security can affect support for clean energy policy. Bernauer and McGrath [17] find that highlighting the potential economic, community building, and health benefits of climate change mitigation does not influence public support for mitigation policies. Myers et al. [18] find framing climate change as a public health issue most effectively elicits hopeful emotions about proposed mitigation policies. Severson and Coleman [19] find that religious moral frames and economic efficiency frames are ineffective in increasing overall support for climate change policies, while secular moral frames, scientific frames, and economic equity frames do boost support. Wiest et al. [20] find that framing climate change as a local or global issue and emphasizing associated losses and benefits can, to a varying degree, affect perceptions of the climate change severity, support for policy action, and behavioral intentions. In sum, the findings of extant studies, while mixed, together imply that certain frames are more likely than others to generate support for climate change.

Taking these varied findings as our point of departure, we aim to contribute to the current discourse by examining how alternative ways of framing climate change affect opinion about climate change policy. Using an experimental approach, we focus on frames that emphasize the putative national security, human rights, and environmental consequences of climate change. We predict that each type of frame should increase the importance that individuals assign to climate change policy, though the effects of the frames will be conditional on partisan attachments and political ideology. In addition, we test whether including the official source of the information provided in the frames conditions their effects. Our findings do not allow us to conclude that issue frames shape the importance assigned to climate change policy in the overall public. However, we do find evidence that Democrats and those on the left sometimes assign more importance to climate change policy when the issue is framed, while, among Republicans and those on the right, certain issue frames lead individuals to rate climate change as *less* important. These patterns tend to be strongest when frames are presented with accompanying sources. We conclude with a brief discussion of our results’ implications for the literature and for the creation of effective policy.

## Expectations about how issue frames affect attitudes about climate policy

Issue framing influences how people process information. In the United States, there is an ongoing debate over the facts of climate change, making framing an attractive method for affecting public opinion about the issue. When the normative foundation surrounding an issue is contested, issue framing is particularly effective [21].

While climate change is most commonly discussed as a threat to the environment, it also has significant implications for national security and human rights. Indeed, security-oriented discussions about climate change were brought up in the 2015 U.S. Department of Defense report on climate change [22]. Additionally, the 2015 White House National Security Strategy identifies climate change as a threat to national security [23]. This suggests that climate change may compete for prominence with other high-salience issues, such as terrorism and economic policy.

How does centralizing security and human rights to the issue of climate change affect public opinion about its importance as a policy issue? Do such frames increase climate change’s salience more than frames that simply reinforce scientific consensus about the issue’s environmental importance? Some insight into these questions can be gained from existing work, which, to a varying degree, has analyzed the effects of security, human rights, and environmental issue frames on a variety of outcomes.

Regarding security frames, Kamradt-Scott and McInnes [24] find that the framing of influenza as a security threat by health practitioners and elected officials increased political attention to an outbreak, motivating policy change. In an experimental setting, Lahav and Courtemanche [25] find that framing immigration as a security threat generates an increase in public support for the limitation of civil liberties. Both studies suggest that individuals become more supportive of a policy when they feel it will mitigate a security risk. As such, we expect that, when climate change is framed as a security threat, individuals will be more likely to want policymakers to tackle the issue. This is expressed in hypothesis form as:

### Hypothesis 1

If climate change is framed as a security risk, then individuals will assign it greater importance.

Several existing studies have examined the relationship between feelings of compassion and support for climate change policies. Lu and Schuldt [26] find that a news article highlighting the impact of an East African drought on a two-year old girl, along with an explicit attribution to climate change, increased climate change policy support among conservatives and moderates. Additionally, Pfattheicher et al. [27] find that compassion for humans is positively related to pro-environmental tendencies. More broadly, the public tends to see human rights as important, and consequently voters hold politicians accountable for their human rights records [28]. We thus expect that a frame that emphasizes the negative consequences of climate change for human rights will lead respondents to assign it greater importance as a policy issue. This is expressed in hypothesis form as:

### Hypothesis 2

If climate change is framed as a human rights risk, then individuals will assign it greater importance.

Fact-based, scientific information can also alter individuals’ attitudes toward salient issues. Aschemann-Witzel and Grunert [29] find that respondents in Denmark and the United States adjust their inferences about the quality of a food supplement when presented with scientific information. Yang and Anderson [30] find that Taiwanese high school seniors adjust their beliefs when confronted with relevant scientific information about nuclear energy’s potential environmental effects. This is consistent with Kolsto’s [31] finding that exposure to scientific information is central to beliefs about the possible relationship between construction of power lines and childhood leukemia. Most pertinent to our study, van der Linden et al. [32] find that the public’s perception of scientific consensus over the causes of climate change is associated with belief in anthropogenic climate change, belief that climate change is a threat, and support for action to mitigate climate change. These studies suggest that opinions about socio-scientific issues are meaningfully updated when individuals are exposed to scientific information. From this, we derive our third hypothesis:

### Hypothesis 3

If climate change is framed in terms of scientific consensus about its environmental consequences, then individuals will assign it greater importance.

While we expect that the overall effect of each frame on the importance individuals assign to climate change policy will be positive, we also recognize that the effects of issue frames can be contingent on political ideology and partisanship. For example, Lahav and Courtemanche [25] find that Democrats are more susceptible to national security frames in regard to attitudes toward immigration, while Gross and D’Ambrosio [33] find that, with regard to emotions experienced, liberals and conservatives react differently to competing frames of the 1992 Los Angeles riots. In Switzerland, Bechtel et al. [34] find that individuals responded to frames about a controversial initiative to deport foreigners convicted of certain crimes by increasing their support for the position that aligns with their partisan affiliation.

In the United States, conservatives and Republicans are relatively likely to see climate change as unimportant and to doubt the veracity of climate science [35, 36]. Gauchat [37] finds that although the public’s trust in science has remained largely stable in the U.S. over the past 40 years, conservatives’ trust in science has dropped within that period. At the same time, Hamilton [38] finds that liberals are more inclined to trust scientific conclusions, even when they conflict with their existing political views. Because information processing is biased toward one’s existing beliefs [39], individuals may construct counterarguments against information that contradicts their preconceptions [40], and they may even bolster their existing beliefs through this counterargumentative process [41]. Hart and Nisbet [42] find evidence of such a dynamic with regard to the likelihood that individuals identify with potential victims of climate change, and Petrovic et al. [15] find similar evidence with regard to the likelihood that individuals agree with a statement about the harmful effects of air pollution. Severson and Coleman [19] and Wiest et al. [20] find that the ideological divide with regard to climate change is strengthened by certain issue frames and attenuated by others.

Kahan et al. [11] advance a “cultural-evaluator model” of risk perception, which puts forth that individuals will weigh the values they share with others like themselves more heavily than potential hazards—in this case, those associated with climate change. Thus, individuals who belong to societal groups predisposed against recognizing the potential ills of climate change will be less likely to change their attitudes when exposed to information about its potential risks. Following from this, we expect that those on the political right and Republicans will be less susceptible to issue frames about the risks associated with climate change. This is expressed in hypothesis form as:

### Hypothesis 4

With regard to the importance assigned to climate change policy, rightists and Republicans will be less susceptible to issue frames than leftists, centrists, Democrats, and independents.

## Experimental protocol

We recruited American adults to our experiment using Amazon’s MTurk in March of 2015. Analyses of MTurk data have been demonstrated to generate valid estimates of treatment effects [43, 44]. However, while MTurk samples in the U.S. are more reflective of the population than other convenience samples [43–46], they are not representative. Thus, our use of MTurk data, while not harmful to internal validity, does affect our ability to make external generalizations.

Payments as low as ten cents have been shown to be sufficient for the collection of high-quality samples on MTurk [44]. We paid participants US$0.30 each for participation, regardless of whether or not they completed the experiment. We randomly assigned participants to a control group or to treatments in which they were asked to read a paragraph-long news story that framed climate change as a national security issue, a human rights issue, or a consequential environmental issue with robust scientific support. The three frames are provided in Table 1, and survey questions and summary statistics are provided in S1 Text and S1 Table. Participants were then asked to complete a short questionnaire. The experiment was administered online using Qualtrics software.

**Table 1:** Text of frames.

Security Frame				
Among the future trends that will impact our national security is climate change. Rising global temperatures, changing precipitation patterns, climbing sea levels, and more extreme weather events will intensify the challenges of global instability, hunger, poverty, and conflict. They will likely lead to food and water shortages, pandemic disease, disputes over refugees and resources, and destruction by natural disasters in regions across the globe. A changing climate will have real impacts on our military and the way it executes its missions. Our armed forces must prepare for a future with a wide spectrum of possible threats, weighing risks and probabilities to ensure that we will continue to keep our country secure. By taking a proactive, flexible approach to assessment, analysis, and adaptation, the Defense Department will keep pace with a changing climate, minimize its impacts on our missions, and continue to protect our national security.				
*Source*: Department of Defense				
Human Rights Frame				
Climate change is a growing concern for the UN refugee agency. In 2013, 22 million people were displaced by disasters brought on by natural hazard events. The Intergovernmental Panel on Climate Change projects an increase in the number of displaced [people] over the course of this century. Climate change will force people into increasing poverty and displacement, rendering both the humanitarian needs and responses in such situations more complex. Scarce natural resources such as drinking water are likely to become even more limited. Many crops and some livestock are unlikely to survive in certain locations if conditions become too hot and dry, or too cold and wet. The adverse effects that climate change may have on natural resources, may spark conflict with other communities, as an increasing number of people compete for a decreasing amount of resources.				
*Source*: United Nations High Commissioner for Refugees				
Environmental Frame				
Science has made enormous progress toward understanding climate change. As a result, there is a strong, credible body of evidence, based on multiple lines of research, documenting that Earth is warming. Global warming is closely associated with other climate changes and impacts, including rising sea levels, increases in intense rainfall events, decreases in snow cover and sea ice, more frequent and intense heat waves, increases in wildfires, longer growing seasons, and ocean acidification. Individually and collectively, these changes pose risks for a wide range of human and environmental systems. Projections of future climate change anticipate an additional warming of 2.0 to 11.5°F (1.1 to 6.4°C) over the 21st century, on top of the 1.4°F already observed over the past 100 years. Furthermore, different sectors, populations, and regions will vary in their exposure and sensitivity to the impacts of these changes.				
*Source*: National Academy of Sciences				

Note: Respondents were randomly assigned to seven groups. The control group did not see a frame. Three groups were treated with one frame each and exposed to the source. Three other groups were treated with one frame each and the source was withheld.

We first requested approval for this experiment and survey from the University of Georgia’s institutional review board, which was granted in Protocol #STUDY00001903. While not written or verbal, participants provided consent online before they read the news stories and were presented with the survey. Before the experiment began, participants were provided with a description of what the experiment would entail, the estimated time the experiment would require, and compensation details. Participants were informed that their MTurk “worker IDs” would not be shared with anyone, would only be collected for the purposes of distributing compensation, and would not be linked with their survey responses. If they consented to undertake the task, participants had the opportunity to click a link that took them to the next screen.

The temporal and geographic distance conveyed in a frame can shape its effect on public opinion. While some studies suggest that localized effects of climate change will most robustly shape individuals’ beliefs about climate change [20, 47, 48], there is also evidence that people perceive climate change as more severe when framed as a geographically and temporally distant phenomenon versus a local phenomenon [49, 50]. In line with the latter findings, our frames conceptualize climate change as a global phenomenon.

Following from Bolsen et al. [51], who find that framing scientific issues in a political light can reduce their effectiveness, we also randomly varied whether or not the source of the frame was presented to participants. As some may see our sources—the U.S. Department of Defense, the United Nations, and the National Academy of Sciences—as political entities, the efficacy of the frames may be reduced when sources are made available. Alternatively, as per Druckman [52], the official nature of these sources may enhance the credibility of the frames, which could potentially make them more effective. As such, we do not have clear expectations about whether the inclusion of sources will make frames more or less effective.

We have 3 (the three different frames) × 2 (sources or no sources) = 6 treatment groups, plus the control group. Our MTurk sample consists of 1104 respondents. Because 51 respondents did not correctly answer a “filter” question and/or failed to answer the question about absolute climate change importance, both of which are provided in S1 Text, we are left with 1053 useable observations (1052 when we consider climate change importance relative to other policy issues). The proportion of respondents correctly answering the filter question does not significantly vary across treatment groups. The number of useable observations per treatment is as follows: Control, 157; Security Frame, No Source, 150; Security Frame, With Source, 153; Human Rights Frame, No Source, 147; Human Rights Frame, With Source, 147; Environmental Frame, No Source, 150; Environmental Frame, With Source, 149.

To examine the randomization process, we use multinomial logit regression to predict one’s assignment to each treatment group as a function of income, gender, education level, political ideology, and partisan affiliation. We could not reject the null hypothesis that each variable’s effect was jointly zero, which suggests that randomization worked. (The *p*-value of a χ^2^ test of the null hypothesis was 0.30 (χ^2^ = 46.19, df = 42).) As such, we are confident that any differences in our outcome variables across treatments are a function of the treatments themselves, rather than differences in the characteristics of participants.

We measure our dependent variable in both absolute and relative terms. For absolute importance, respondents were asked to assign a value from 0 to 10 to the importance of climate change policy, with zero representing no importance and ten representing very high importance. Relative importance is measured as the rank that the respondents assign to climate change among five other salient global policy issues: democratization, public health, international economic policy, the spread of nuclear weapons, and terrorism. These issues were sourced from a Gallup poll [53], a Pew Research Center poll [54], and the Eurobarometer survey [55]. We see utility in examining both absolute and relative importance, as an individual could rank an issue as having high absolute importance, but rank the same issue low in comparison to other issues perceived as salient.

## Results

Because, as described above, assignment to the control and treatment groups appears to be truly random, we examine the effects of the treatments using a difference-in-means estimator. Here, we convey the results of these tests graphically, and we provide corresponding numerical information in S2 Table and S3 Table. In all subsequent figures, a confidence interval that overlaps 0 indicates that a particular treatment effect is not significant at the 0.10 level, two-sided.

**Fig 1:**
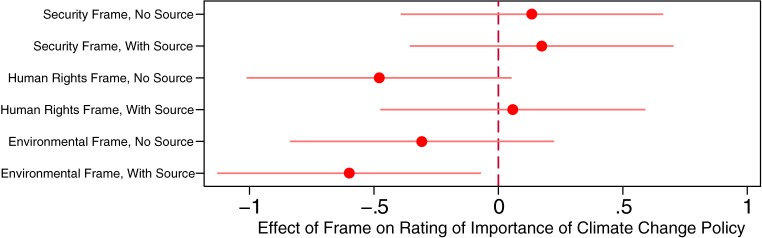
Climate change frames and ratings of the importance of climate change policy. Note: Horizontal bars indicate 90% confidence intervals.

We first focus on individuals’ opinions about the absolute importance of climate change policy. Fig 1 reports differences in mean ratings of the importance of climate change policy across the six treatments. Differences are considered relative to the control group, in which the mean rating of climate change importance on the 0–10 scale is 7.14. We do not observe a statistically significant positive effect of framing in any of the treatments, which works against our first three hypotheses. In fact, on average, those treated with the environmental frame who saw the source rate climate change policy as significantly *less* important (*p =* 0.063, two sided).

**Fig 2:**
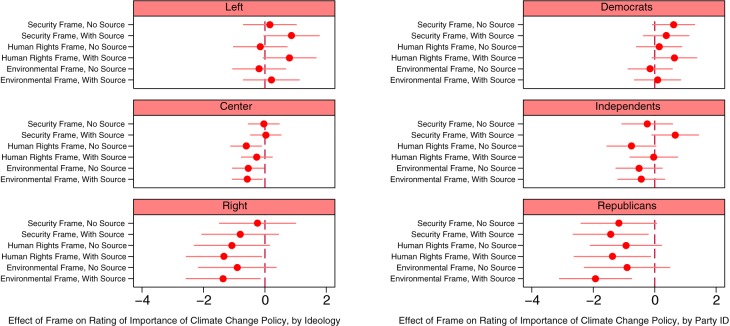
Climate change frames and ratings of the importance of climate change policy, over ideology and partisanship. Note: Horizontal bars indicate 90% confidence intervals. “Left” is those who reported a 0–10 ideology of 0, “Center” is 5, and “Right” is 10.

These generally null findings may arise because the treatment effects are heterogeneous in the sample and cancel out in the aggregate; as per our fourth hypothesis, the effects of issue frames may vary according to political ideology and partisan attachments. In particular, we expect that framing effects will be weakest for those on the right and Republicans. In Fig 2, we present the effects of our treatments across ideological and partisan groups. Because of missing data, the number of observations drops by two when party identification is included in the analyses and by three when ideology is included. As shown in the top two panels of the figure, while effects tend to be statistically insignificant or marginally significant, the results do suggest that those on the political left and Democrats are relatively likely to respond positively to the frames, especially those with accompanying sources.

Rather than being unaffected by frames, those on the right and Republicans tend to rate climate change policy as *less* important than their counterparts in the control group when the issue is framed. This is especially true when sources are provided. For example, as shown in the bottom-right panel of Fig 2, as compared to those in the control group, Republicans who are exposed to sourced issue frames, on average, rate the importance of climate change policy about 1.5–2.0 points lower on the 0–10 scale. Sourced frames produce a “boomerang” or “backfire” effect among Republicans. The negative effect is strongest in the environmental frame, in which the source is the National Academy of Sciences. There is also some evidence of “backfire” among centrists and independents, though, even when statistically significant, effect sizes are relatively small.

**Fig 3:**
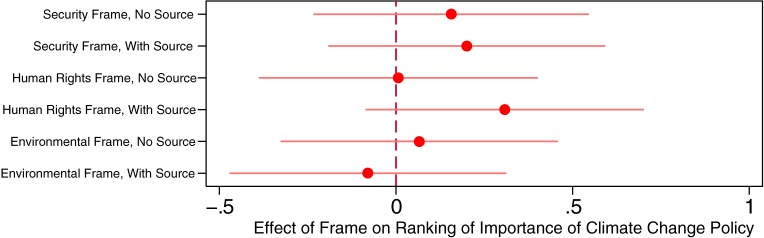
Climate change frames and rankings of the importance of climate change policy. Note: Horizontal bars indicate 90% confidence intervals.

We next consider the measure of relative climate change policy importance, in which climate change policy importance is ranked relative to the five other major global issues. The measure ranges from 1 to 6, and we reverse the scale so higher values mean more importance. In the control group, the mean reversed ranking of climate change is 3.73. Fig 3 illustrates the differences in the mean rankings of the importance of climate change policy across the six treatments, relative to the control group. Similar to when absolute ratings were taken as the dependent variable, we do not observe a statistically significant effect of framing on rankings of the importance of climate change in any of the treatments, which again works against our first three hypotheses.

**Fig 4:**
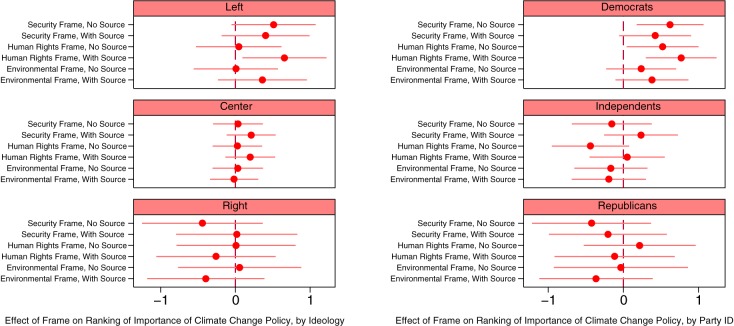
Climate change frames and rankings of the importance of climate change policy, over ideology and partisanship. Note: Horizontal bars indicate 90% confidence intervals. “Left” is those who reported a 0–10 ideology of 0, “Center” is 5, and “Right” is 10.

Fig 4 illustrates how the effects of issue frames on relative rankings of climate change policy importance vary across ideological and partisan groups. Here we again find some support for Hypothesis 4. As shown in the upper-right panel of Fig 4, Democrats respond positively to many of the frames, and the sourced human rights frame has the strongest effect; Democrats who are exposed to the sourced human rights frame rank climate change policy nearly a full place above those in the control group. The upper-left panel of the figure suggests similar patterns among those on the political left. The remaining panels of the figure demonstrate that, with regard to opinions about the relative importance of climate change policy, we find no evidence that issue frames affect independents, centrists, Republicans, and rightists.

## Discussion

We find little evidence that framing climate change as a matter of national security, a human rights issue, or a problem of environmental consequence alters overall perceptions of its importance as a policy issue. Still, our null findings do not necessarily indicate that issue frames have no effect. That is, our inability to reject the null hypotheses of no treatment effects only indicates that we cannot conclude that there are treatment effects; it does not allow us to conclude that there are *no* treatment effects [56]. Further, this is not the first study of opinions about climate change to find unexpected results [57]. In fact, our finding that frames have little overall effect on climate opinion are consistent with those of Bernauer and McGrath [17], but it is worth exploring why we find mostly null results where other experimental examinations largely have not.

One potential answer lies in the nature of our treatments, as compared to those previously employed, Aklin and Urpelainen also test a security frame [16], but it is much shorter than ours. Myers et al. [18] also examine environmental and national security frames, but they do so in the context of longer newspaper articles. Severson and Coleman [19] also examine scientific environmental frames, but they were conveyed with vignettes containing short arguments. And, the frames of Kahan et al. [11], Yarnal et al. [13], Schuldt et al. [14], Petrovic et al. [15], and Wiest et al. [20] have substantially different content than our own.

Further, all three of our frames treat climate change as a global phenomenon and discuss the impacts of climate change at the global level. While Leiserowitz [49] and Räthzel and Uzzell [50] find that individuals see climate change to be more severe at the global level than at the local level, climate change may become a psychologically abstract event when placed in a geographically distant context. As individuals have more difficulty evaluating and making decisions about psychologically abstract concepts than about more concrete concepts [58, 59], the global scale of our frames could dampen the level of importance individuals assign to climate change. Indeed, a number of studies suggest that local frames may have particularly strong effects [20, 47, 48]. Wiest et al. [20], in particular, find that local frames increase perceptions of severity and support for localized policies regardless of partisanship—and change behavioral intentions among Republicans and independents.

Our findings may also differ from those of extant experimental work due to sample dissimilarities. While Aklin and Urpelainen [16] Petrovic et al. [15], Myers et al. [18], and Severson and Coleman [19] also use random samples of American adults, Kahan et al. [11] examine individuals from both the U.S. and England, Yarnal et al. [13] only sample individuals from central Pennsylvania, Schuldt et al. [14] use respondents who routinely complete a monthly 30-minute survey session as part of the University of Michigan Survey of Consumer Attitudes, and Wiest et al. [20] use a sample of university students.

Finally, our findings may differ from previous studies because we analyze different outcome variables. While we focus on the absolute and relative importance that individuals assign to climate change policy, Yarnal et al. [13] consider support for various government programs aimed at addressing climate change and the likelihood that one will take voluntary action to combat it, Schuldt et al. [14] examine whether individuals believe the climate is actually changing, Aklin and Urpelainen [16] examine support for clean energy policy alone, Petrovic et al. [15] examine whether individuals agree that air pollution is harmful, Kahan et al. [11] examine whether individuals found published studies about climate change valid and their beliefs about extent to which climate change is occurring, and Myers et al. [18] examine emotional reactions. Severson and Coleman’s [19] outcome variable, support for climate change mitigation policies, is closest to ours. Wiest et al. [20] also employ support for policy action as one of their three outcome variables.

In part because it differs from previous examinations, our experiment makes novel contributions to the literature. First, we analyze the effects of three very different frames, which helps us determine whether it is the simple presence of frames or their content that changes public opinion. We find little evidence that the content of frames matters. Second, we conduct a novel examination of the effects of frames with and without their official sources. We find that, especially among Republicans, frames have a larger (and negative) effect on the importance individuals assign to climate change policy when sources are included. Third, we focus on both the absolute and relative importance that individuals assign to climate change policy. We find that Republicans, independents, and political rightists and centrists have more resolute opinions about relative importance, while Democrats, independents, and political leftists and centrists have more resolute opinions about absolute importance. Fourth, we test for potential conditioning effects of both political ideology and partisanship, and evidence suggests that the latter is a more robust conditioner of the impact of issue frames.

Hochschild and Einstein [60] discuss how misinformation can produce bad policies, as politicians often cater to the preferences of those who hold mistaken beliefs about climate science, and thus do not see value in addressing the issue. For those would like to induce public concern about climate change by “repackaging” the issue, the results of this study are potentially discouraging: our findings suggest that, on average, individuals’ beliefs about the importance of climate change are quite rigid, and those seeking to alter such beliefs will find little utility in issue framing.

## Supporting information

S1 Text Survey questions. (DOCX)

S1 Table Summary statistics. (DOCX)

S2 Table Climate change frames and ratings of the importance of climate change policy. Note: Top section of table corresponds with Fig 1; bottom six sections correspond with Fig 2.
(DOCX)

S3 Table Climate change frames and rankings of the importance of climate change policy. Note: Top section of table corresponds with Fig 3; bottom six sections correspond with Fig 4.
(DOCX)
