# Supplementary material for: How issue frames shape beliefs about the importance of climate change policy across ideological and partisan groups
Source: PLoS One. 2017 Jul 20;12(7):e0181401. doi: 10.1371/journal.pone.0181401 (PMC5519075; doi:10.1371/journal.pone.0181401)
Supplement: S1 Text — (DOCX) [file pone.0181401.s001.docx]

**S1 Text. Survey Questions.**

**Rank the following policy issues in order of importance from 1 (most important) to 6 (least important):**

Listed in alphabetical order

____ Climate change

____ Democratization

____ Global public health

____ International economic policy

____ Spread of nuclear weapons

____ Terrorism

**How important is climate change as policy issue, where 0 means not important at all and 10 means very important?**

(Not important at all) 0 1 2 3 4 5 6 7 8 9 10 (Extremely important)

What is your gender?:

- Female (1)
- Male (0)

**In what year were you born?:**

**What is your highest level of education completed?:**

- Some high school
- High school
- Some college
- Trade/technical/vocational training
- Bachelor’s degree
- Master’s degree
- Ph. D +

**Generally speaking, do you think of yourself as a Republican, Democrat, independent, or something else?**

- Republican
- Democrat
- Independent
- Something Else

**If Republican: Would you call yourself a strong Republican or a not very strong Republican?**

- Strong Republican
- Not Very Strong Republican

**If Democrat: Would you call yourself a strong Democrat or a not very strong Democrat?**

- Strong Democrat
- Not Very Strong Democrat

**If Independent: Do you think of yourself as closer to the Republican Party or Democratic Party?**

- Lean Republican
- Lean Democrat
- Neither

**In politics people sometimes talk of left and right. Where would you place yourself on a scale from 0 to 10 where 0 means the left and 10 means the right?**

(Left) 0 1 2 3 4 5 6 7 8 9 10 (Right)

**In what state do you live?**

**What is your average annual household income before tax:**

- $24,999 or less
- $25,000-$49,999
- $50,000-$99,999
- $100,000 and up

**Research in decision making shows that people, when making decisions and answering questions, prefer not to pay attention and minimize their effort as much as possible. Some studies show that over 50% of people don’t carefully read questions. If you are reading this question and have read all the other questions, please select the box marked “other”. Do not select “climate policy.” Thank you for participating and taking the time to read through the questions carefully! What was this study about?**

- Domestic Politics
- Climate Policy
- The European Union
- Other
