# Supplementary material for: How issue frames shape beliefs about the importance of climate change policy across ideological and partisan groups
Source: PLoS One. 2017 Jul 20;12(7):e0181401. doi: 10.1371/journal.pone.0181401 (PMC5519075; doi:10.1371/journal.pone.0181401)
Supplement: S1 Table — (DOCX) [file pone.0181401.s002.docx]

| **S1 Table. Summary Statistics.** | | | | | |
| --- | --- | --- | --- | --- | --- |
|  |  |  |  |  | |
|  | **Mean** | **Std. Dev.** | **Min.** | **Max.** | |
| Rating of Important of Climate Change | 7.01 | 2.83 | 0 | | 10 |
| Ranking of Important of Climate Change (six = most important) | 3.18 | 1.75 | 1 | | 6 |
| Income | 2.28 | 0.95 | 1 | | 4 |
| Female | 0.35 | 0.48 | 0 | | 1 |
| Education Level | 4.12 | 1.30 | 1 | | 7 |
| Age | 38.96 | 18.49 | 19 | | 96 |
| Ideology | 3.96 | 2.59 | 0 | | 10 |
| Democrat | 0.44 | 0.50 | 0 | | 1 |
| Independent | 0.39 | 0.49 | 0 | | 1 |
| Republican | 0.17 | 0.38 | 0 | | 1 |
|  |  |  |  |  | |
