# Supplementary material for: How issue frames shape beliefs about the importance of climate change policy across ideological and partisan groups
Source: PLoS One. 2017 Jul 20;12(7):e0181401. doi: 10.1371/journal.pone.0181401 (PMC5519075; doi:10.1371/journal.pone.0181401)
Supplement: S2 Table — Note: Top section of table corresponds with Fig 1; bottom six sections correspond with Fig 2. (DOCX) [file pone.0181401.s003.docx]

| **S2 Table. Climate Change Frames and Ratings of the Importance of Climate Change Policy.** | | | |
| --- | --- | --- | --- |
|  |  |  |  |
|  | **Estimated Treatment Effect** | **Two-Sided *p*-value** | **Post-Hoc Power** |
|  |  |  |  |
| Security Frame, No Source | 0.135 | 0.674 | 0.110 |
| Security Frame, With Source | 0.174 | 0.589 | 0.135 |
| Human Rights Frame, No Source | -0.479 | 0.139 | 0.435 |
| Human Rights Frame, With Source | 0.058 | 0.858 | 0.071 |
| Environmental Frame, No Source | -0.307 | 0.341 | 0.244 |
| Environmental Frame, With Source | -0.600 | 0.063 | 0.586 |
|  |  |  |  |
| **Left** |  |  |  |
| Security Frame, No Source | 0.161 | 0.762 | 0.090 |
| Security Frame, With Source | 0.867 | 0.119 | 0.467 |
| Human Rights Frame, No Source | -0.150 | 0.781 | 0.086 |
| Human Rights Frame, With Source | 0.803 | 0.131 | 0.447 |
| Environmental Frame, No Source | -0.187 | 0.725 | 0.098 |
| Environmental Frame, With Source | 0.211 | 0.707 | 0.102 |
|  |  |  |  |
| **Center** |  |  |  |
| Security Frame, No Source | -0.039 | 0.901 | 0.064 |
| Security Frame, With Source | 0.031 | 0.920 | 0.061 |
| Human Rights Frame, No Source | -0.612 | 0.050 | 0.623 |
| Human Rights Frame, With Source | -0.265 | 0.400 | 0.211 |
| Environmental Frame, No Source | -0.543 | 0.089 | 0.524 |
| Environmental Frame, With Source | -0.574 | 0.060 | 0.595 |
|  |  |  |  |
| **Right** |  |  |  |
| Security Frame, No Source | -0.239 | 0.754 | 0.092 |
| Security Frame, With Source | -0.805 | 0.293 | 0.276 |
| Human Rights Frame, No Source | -1.073 | 0.153 | 0.414 |
| Human Rights Frame, With Source | -1.332 | 0.078 | 0.549 |
| Environmental Frame, No Source | -0.899 | 0.248 | 0.313 |
| Environmental Frame, With Source | -1.359 | 0.067 | 0.576 |
|  |  |  |  |
| **Democrats** |  |  |  |
| Security Frame, No Source | 0.613 | 0.150 | 0.419 |
| Security Frame, With Source | 0.375 | 0.413 | 0.205 |
| Human Rights Frame, No Source | 0.145 | 0.750 | 0.092 |
| Human Rights Frame, With Source | 0.641 | 0.155 | 0.413 |
| Environmental Frame, No Source | -0.147 | 0.740 | 0.095 |
| Environmental Frame, With Source | 0.094 | 0.839 | 0.075 |
|  |  |  |  |
| **Independents** |  |  |  |
| Security Frame, No Source | -0.242 | 0.633 | 0.121 |
| Security Frame, With Source | 0.666 | 0.155 | 0.412 |
| Human Rights Frame, No Source | -0.756 | 0.124 | 0.458 |
| Human Rights Frame, With Source | -0.030 | 0.950 | 0.057 |
| Environmental Frame, No Source | -0.509 | 0.274 | 0.291 |
| Environmental Frame, With Source | -0.437 | 0.354 | 0.236 |
|  |  |  |  |
| **Republicans** |  |  |  |
| Security Frame, No Source | -1.167 | 0.123 | 0.459 |
| Security Frame, With Source | -1.433 | 0.056 | 0.605 |
| Human Rights Frame, No Source | -0.935 | 0.189 | 0.371 |
| Human Rights Frame, With Source | -1.379 | 0.071 | 0.563 |
| Environmental Frame, No Source | -0.899 | 0.291 | 0.278 |
| Environmental Frame, With Source | -1.926 | 0.008 | 0.848 |
|  |  |  |  |
| Note: Top section of table corresponds with Fig 1; bottom six sections correspond with Fig 2. | | | |
