# Supplementary material for: How issue frames shape beliefs about the importance of climate change policy across ideological and partisan groups
Source: PLoS One. 2017 Jul 20;12(7):e0181401. doi: 10.1371/journal.pone.0181401 (PMC5519075; doi:10.1371/journal.pone.0181401)
Supplement: S3 Table — Note: Top section of table corresponds with Fig 3; bottom six sections correspond with Fig 4. (DOCX) [file pone.0181401.s004.docx]

| **S3 Table. Climate Change Frames and Rankings of the Importance of Climate Change Policy.** | | | |
| --- | --- | --- | --- |
|  |  |  |  |
|  | **Estimated Treatment Effect** | **Two-Sided *p*-value** | **Post-Hoc Power** |
|  |  |  |  |
| Security Frame, No Source | 0.156 | 0.432 | 0.195 |
| Security Frame, With Source | 0.201 | 0.316 | 0.261 |
| Human Rights Frame, No Source | 0.007 | 0.973 | 0.054 |
| Human Rights Frame, With Source | 0.308 | 0.125 | 0.456 |
| Environmental Frame, No Source | 0.066 | 0.742 | 0.094 |
| Environmental Frame, With Source | -0.079 | 0.691 | 0.106 |
|  |  |  |  |
| **Left** |  |  |  |
| Security Frame, No Source | 0.512 | 0.134 | 0.442 |
| Security Frame, With Source | 0.404 | 0.258 | 0.304 |
| Human Rights Frame, No Source | 0.045 | 0.896 | 0.065 |
| Human Rights Frame, With Source | 0.658 | 0.055 | 0.609 |
| Environmental Frame, No Source | 0.007 | 0.984 | 0.052 |
| Environmental Frame, With Source | 0.363 | 0.316 | 0.260 |
|  |  |  |  |
| **Center** |  |  |  |
| Security Frame, No Source | 0.035 | 0.861 | 0.071 |
| Security Frame, With Source | 0.211 | 0.290 | 0.279 |
| Human Rights Frame, No Source | 0.027 | 0.892 | 0.066 |
| Human Rights Frame, With Source | 0.199 | 0.325 | 0.254 |
| Environmental Frame, No Source | 0.032 | 0.874 | 0.069 |
| Environmental Frame, With Source | -0.017 | 0.933 | 0.059 |
|  |  |  |  |
| **Right** |  |  |  |
| Security Frame, No Source | -0.441 | 0.369 | 0.228 |
| Security Frame, With Source | 0.018 | 0.971 | 0.054 |
| Human Rights Frame, No Source | 0.009 | 0.985 | 0.052 |
| Human Rights Frame, With Source | -0.259 | 0.593 | 0.133 |
| Environmental Frame, No Source | 0.058 | 0.908 | 0.063 |
| Environmental Frame, With Source | -0.396 | 0.407 | 0.208 |
|  |  |  |  |
| **Democrats** |  |  |  |
| Security Frame, No Source | 0.622 | 0.021 | 0.745 |
| Security Frame, With Source | 0.422 | 0.147 | 0.424 |
| Human Rights Frame, No Source | 0.522 | 0.073 | 0.560 |
| Human Rights Frame, With Source | 0.769 | 0.007 | 0.852 |
| Environmental Frame, No Source | 0.237 | 0.401 | 0.210 |
| Environmental Frame, With Source | 0.380 | 0.198 | 0.361 |
|  |  |  |  |
| **Independents** |  |  |  |
| Security Frame, No Source | -0.154 | 0.634 | 0.121 |
| Security Frame, With Source | 0.235 | 0.430 | 0.196 |
| Human Rights Frame, No Source | -0.436 | 0.163 | 0.402 |
| Human Rights Frame, With Source | 0.050 | 0.868 | 0.070 |
| Environmental Frame, No Source | -0.165 | 0.576 | 0.139 |
| Environmental Frame, With Source | -0.193 | 0.519 | 0.159 |
|  |  |  |  |
| **Republicans** |  |  |  |
| Security Frame, No Source | -0.423 | 0.379 | 0.222 |
| Security Frame, With Source | -0.206 | 0.666 | 0.113 |
| Human Rights Frame, No Source | 0.217 | 0.630 | 0.122 |
| Human Rights Frame, With Source | -0.116 | 0.811 | 0.080 |
| Environmental Frame, No Source | -0.033 | 0.952 | 0.057 |
| Environmental Frame, With Source | -0.363 | 0.428 | 0.197 |
|  |  |  |  |
| Note: Top section of table corresponds with Fig 3; bottom six sections correspond with Fig 4. | | | |
